# Vascular endothelial cells cultured from patients with cerebral or uncomplicated malaria exhibit differential reactivity to TNF

**DOI:** 10.1111/j.1462-5822.2010.01528.x

**Published:** 2011-02

**Authors:** Samuel Crocodile Wassmer, Christopher Alan Moxon, Terrie Taylor, Georges Emile Grau, Malcolm Edward Molyneux, Alister Gordon Craig

**Affiliations:** 1 Malawi-Liverpool-Wellcome Trust Clinical Research ProgrammeCollege of Medicine, PO Box 30096 Chichiri, Blantyre 3, Malawi; 2 Liverpool School of Tropical MedicineLiverpool L3 5QA, UK; 3 Department of Internal Medicine, College of Osteopathic Medicine, Michigan State UniversityEast Lansing, MI 48824, USA; 4 Blantyre Malaria ProjectPO Box 32256 Chichiri, Bantyre 3, Malawi; 5 Vascular Immunology Unit, Department of Pathology, Bosch Institute, Sydney Medical School, The University of SydneySydney, NSW 2402, Australia

## Abstract

*Plasmodium falciparum* malaria is a major cause of morbidity and mortality in African children, and factors that determine the development of uncomplicated (UM) versus cerebral malaria (CM) are not fully understood. We studied the *ex vivo* responsiveness of microvascular endothelial cells to pro-inflammatory stimulation and compared the findings between CM and UM patients. In patients with fatal disease we compared the properties of vascular endothelial cells cultured from brain tissue to those cultured from subcutaneous tissue, and found them to be very similar. We then isolated, purified and cultured primary endothelial cells from aspirated subcutaneous tissue of patients with CM (EC^CM^) or UM (EC^UM^) and confirmed the identity of the cells before analysis. Upon TNF stimulation *in vitro*, EC^CM^ displayed a significantly higher capacity to upregulate ICAM-1, VCAM-1 and CD61 and to produce IL-6 and MCP-1 but not RANTES compared with EC^UM^. The shedding of endothelial microparticles, a recently described parameter of severity in CM, and the cellular level of activated caspase-3 were both significantly greater in EC^CM^ than in EC^UM^. These data suggest that inter-individual differences in the endothelial inflammatory response to TNF may be an additional factor influencing the clinical course of malaria.

## Introduction

Cerebral malaria (CM) is an important life-threatening complication of *Plasmodium falciparum* infection. This neurological syndrome occurs in about 1% of infections but exhibits significant case fatality rates, contributing up to a million deaths annually among children in sub-Saharan Africa (Rowe *et al*., 2006). The pathophysiological mechanisms underlying CM are not fully understood and several hypotheses have been put forward, including mechanical obstruction of microvessels by *P. falciparum*-parasitized red blood cells (PRBC) and hyper-activation of host immune cells leading to the excessive release of pro-inflammatory cytokines (Berendt *et al*., 1994; Clark and Rockett, 1994; Grau and de Kossodo, 1994). These complementary theories may contribute to our understanding of pathogenesis, but they fail to explain why only a small percentage of malaria patients develop CM.

Sequestration of PRBC within brain microvasculature is a common feature of CM, and this phenomenon is mediated by several endothelial cell (EC) adhesion molecules including inter-cellular adhesion molecule (ICAM)-1, CD36, P-selectin and vascular cell adhesion molecule (VCAM)-1 (Ho and White, 1999). Most of these receptors are inducible in endothelium by pro-inflammatory cytokines such as TNF, a cytokine that has been postulated to play a crucial role in the pathogenesis of CM (Grau *et al*., 1989; Hunt and Grau, 2003). In *P. falciparum*-infected patients, the surface expression of vascular endothelial receptorshas been shown to be increased in several tissues at *post-mortem*, including the brain. A colocalization of PRBC and these receptors has also been observed, suggesting that endothelial activation plays a role in sequestration, and therefore potentially in pathogenesis (MacPherson *et al*., 1985; Turner *et al*., 1994). In addition to the upregulation of endothelial receptors mediating PRBC sequestration, TNF induces extensive alterations in microvascular EC including morphological reorganization (Pober and Cotran, 1990), release of membrane microparticles (MP) (Zwaal and Schroit, 1997), production of pro-inflammatory cytokines and apoptosis (Grell *et al*., 1999; Gnant *et al*., 2000; Kimura *et al*., 2003).

In an experimental model for CM, it has been reported that brain EC purified from CM-susceptible and CM-resistant mice exhibit different sensitivities to TNF. Brain EC isolated from susceptible mice had a greater capacity to produce interleukin (IL)-6 and to upregulate ICAM-1 and VCAM-1 in response to TNF than did brain EC from resistant mice (Lou *et al*., 1998). On this basis, we postulated that differences in the severity of human malarial disease might in part be due to a differential responsiveness of the host endothelium to systemic inflammation.

We studied the effect of TNF on cultured EC originating from Malawian children with different clinical syndromes. We compared upregulation of cytoadherence receptors, shedding of MP, release of chemokines and cytokines selected for their relevance in the pathophysiology of malaria and activation of pro-apoptotic factors in EC cultured from patients in the different clinical groups. Since the isolation, purification and culture of brain vascular EC is feasible only through *post-mortem* sampling, we used subcutaneous adipose tissue as a source of vascular EC from all patient groups. Wilairatana and colleagues described focal cytokine accumulations in the skin that were similar to those in the brain (Wilairatana *et al*., 2000). This was recently confirmed in humans, where the subcutaneous adipose tissue, but not dermal or epidermal skin layers, was a major site for sequestration (Seydel *et al*., 2006). Nakano and colleagues reported a correlation between PRBC sequestration in subcutaneous tissues and in brain microvasculature in a primate model of CM (Nakano *et al*., 1996).

In view of these data, and considering that purification and isolation of fat-derived EC has been extensively described and can be performed routinely in living patients (Kern *et al*., 1983), EC derived from human subcutaneous fat appeared to represent an original and relevant alternative model to test our hypothesis and compare endothelial reactivity to TNF in patients with uncomplicated malaria (UM) and CM.

## Results

### Success rates of brain and subcutaneous fat cell preparations

The success rate of skin cell preparation from tissue samples was of 73.3%, with six successful preparations out of eight UM patients and five out of eight CM patients respectively. In the case of brain cell preparations, we obtained six cell cultures out of the same eight patients (75% of success) but restricted our analyses to the five patients we successfully isolated, purified and cultured both brain and fat EC. The loss of approximately 25% of our samples in both patient categories was mainly due to either bacterial or fungal contamination, a risk considerably increased by the collection of tissue samples in a non-sterile environment (i.e. ward or mortuary). In one case of brain EC preparation, cells stopped growing after 2 days in culture with no known explanation.

### Comparison of morphological and phenotypical characteristics between subcutaneous fat and brain-derived EC^CM^

**Fig. 1:**
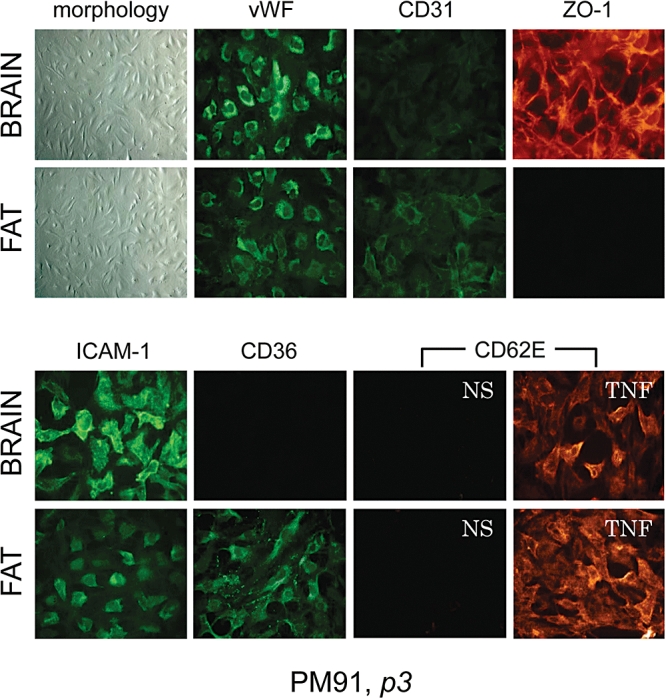
Immunofluorescence analysis of endothelial markers on subcutaneous fat and brain-derived EC^CM^ monolayers. The figure shows evidence for the expression of several typical endothelial markers on submembrane or cell type surface, including vWF, CD31, ZO-1, ICAM-1, CD36 and CD62-E. Micrographs presented here are from patient PM91-derived cells in *passage 3* and are representative of the results obtained with all the other CM patients. Magnification: × 100 for general morphology micrographs, × 400 for immunofluorescence micrographs.

Brain and subcutaneous fat-derived primary EC^CM^ readily adhere to plastic substrate, form flat monolayers after plating in culture flasks, present a thin cobblestone-like morphology with central and prominent nuclei after 3 days in culture, and exhibit contact inhibition (Fig. 1). All of the primary EC^CM^ constitutively expressed typical endothelial markers including von Willebrand factor (vWF) and platelet-endothelium cell adhesion molecule (PECAM)-1 (CD31), and showed an E-selectin (CD62E) inducible expression upon TNF stimulation, as shown in Fig. 1 and Table 1. Brain and subcutaneous fat EC constitutively expressed high levels of ICAM-1 (Fig. 1), αvβ3 integrin (CD51/CD61), CD146 and VCAM-1, and both ICAM-1 and VCAM-1 were significantly upregulated upon TNF stimulation (Table 1). However, brain-derived primary EC exhibited a peripheral pattern of positive staining for ZO-1, a constituent of endothelial tight junctions, as well as no CD36 expression, whereas subcutaneous fat EC did not express ZO-1 but were stained positively for CD36 (Fig. 1).

**Table 1:** Flow cytometric phenotyping of EC^CM^ under resting and activated conditions

			Subcutaneous fat EC^CM^				Brain EC^CM^			
				
			Resting		TNF		Resting		TNF	
						
CD	Antigen	Clone	MFI (a.u.)	% POS	MFI (a.u.)	% POS	MFI (a.u.)	% POS	MFI (a.u.)	% POS
IgG1			0.23		0.21		0.19		0.22	
CD31	PECAM-1	5.6E	30	89	34.7	78.9	25.9	78.8	27.3	81.4
CD36	GPIV	FA6-152	37.2	48.2	40.3	51	0.23	0.7	0.26	0.9
CD40	Bp50	B-B20	0.42	2.8	0.46	6.1	0.24	3.5	0.6	6.3
CD51	αv	AMF7	20.2	100	19.7	100	24.3	100	25.8	100
CD54	ICAM-1	84H10	43.8	94.7	84.6	96	35	92.7	79.8	90.6
CD61	β3	SZ21	2.1	100	34.1	100	3.7	100	31.6	100
CD62E	E-selectin	CL2/6	0.26	2	0.32	2.7	0.4	1.3	0.22	0.7
CD106	VCAM-1	1G1	5.6	80	27.1	78.3	4.1	82.6	24.7	81.1
CD146	MCAM	OJ79c	28.1	76	25.9	72.7	23	89.3	21.5	92.1

Subcutaneous fat- and brain-derived EC isolated from patients who died of CM were either left unstimulated or activated with TNF (10 ng ml^−1^) and stained by indirect immunolabelling after 12 h, except for CD62E, which was studied after 6 h. Results are representative of a series of three experiments performed in *passage 2* and show both the mean fluorescence intensity (MFI) expressed in arbitrary units (a.u.) of the labelling with specific monoclonal antibodies and also the percentage of cells positively labelled with these antibodies in the whole population.

### Variation of ICAM-1, VCAM-1 and CD61 upregulation in subcutaneous fat EC^CM^ and EC^UM^ in the presence of a pro-inflammatory stimulus

**Fig. 2:**
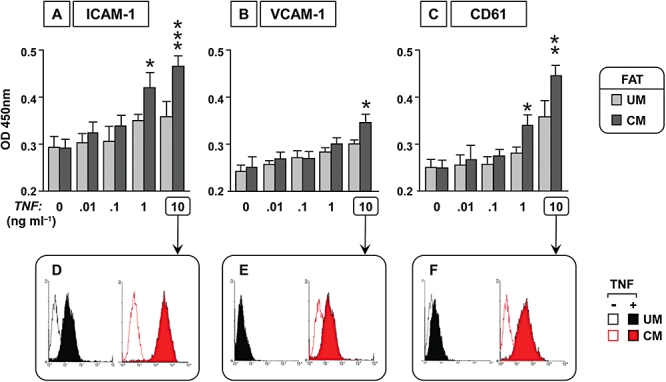
Comparison of TNF-induced adhesion molecule upregulation between EC^UM^ and EC^CM^ of subcutaneous fat origin. Cells were seeded in a 96-well plate and stimulated by increasing concentrations of TNF. EC^UM^ and EC^CM^ were then fixed and the expression of ICAM-1, VCAM-1 and CD61 was measured by cell-based ELISA (A, B and C respectively). Results are expressed as mean ± SD of two experiments (Dunn's test, *P < 0.05, **P < 0.01 and ***P < 0.001). As a control, each experiment was confirmed by flow cytometry (D–F). The results of one representative reading obtained with the greatest concentration of TNF are shown here.

Under resting conditions, the basal expression of ICAM-1, VCAM-1 and CD61 did not show any significant difference between EC^CM^ and EC^UM^ in cell-based ELISA assays using subcutaneous fat ECs. In contrast, upon overnight stimulation with increasing doses of TNF, cells of both patient categories exhibited a dose-dependent upregulation of the three surface molecules. When the dose of TNF reached a range between 1 and 10 ng ml^−1^, a significantly higher upregulation of ICAM-1 (*P* < 0.05 and *P* < 0.001, respectively, Fig. 2A) and CD61 (*P* < 0.05 and *P* < 0.01, respectively, Fig. 2C) was observed in EC^CM^ compared with EC^UM^. In the case of VCAM-1, a significant difference was only observed at a TNF concentration of 10 ng ml^−1^ (*P* < 0.05, Fig. 2B). All these results were confirmed by flow cytometry at the highest TNF concentration (10 ng ml^−1^, Fig. 2D–F). Brain-derived EC^CM^ were also used as a comparator and when stimulated with the most effective dose of TNF no differences were observed in terms of ICAM-1, VCAM-1 and CD61 upregulation, when compared with subcutaneous fat-derived EC^CM^ (data not shown).

### Shedding of endothelial membrane MP

**Fig. 3:**
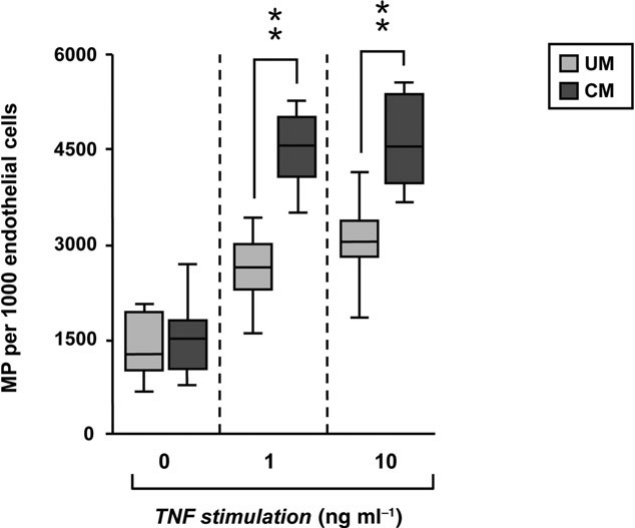
Quantification of TNF-induced endothelial MP release by subcutaneous fat-derived EC^UM^ and EC^CM^. Cells were cultured and left resting or stimulated with TNF for 6 h before analysis. MP production was quantified by flow cytometry for each condition. Results (two determinations in three experiments) are expressed in numbers of MP labelled with annexin V-FITC, extracted from culture supernatants of 1000 EC (Dunn's test, **P < 0.01).

EC^CM^ and EC^UM^ were exposed to two concentrations of TNF, for which significant effects have been observed in previous assays (1 and 10 ng ml^−1^ respectively), to compare MP formation. Both cell types produced significantly higher MP numbers than in resting conditions and a dose-dependent increase of MP production was observed (Fig. 3). In both stimulation conditions the number of MP released by 1000 EC was significantly higher in EC^CM^ than in EC^UM^ (*P* < 0.01), EC^CM^ releasing up to 5420 MP per 1000 EC when stimulated with 10 ng ml^−1^ TNF (Fig. 3).

### MCP-1, RANTES and IL-6 release by EC^CM^ and EC^UM^ upon TNF stimulation

**Fig. 4:**
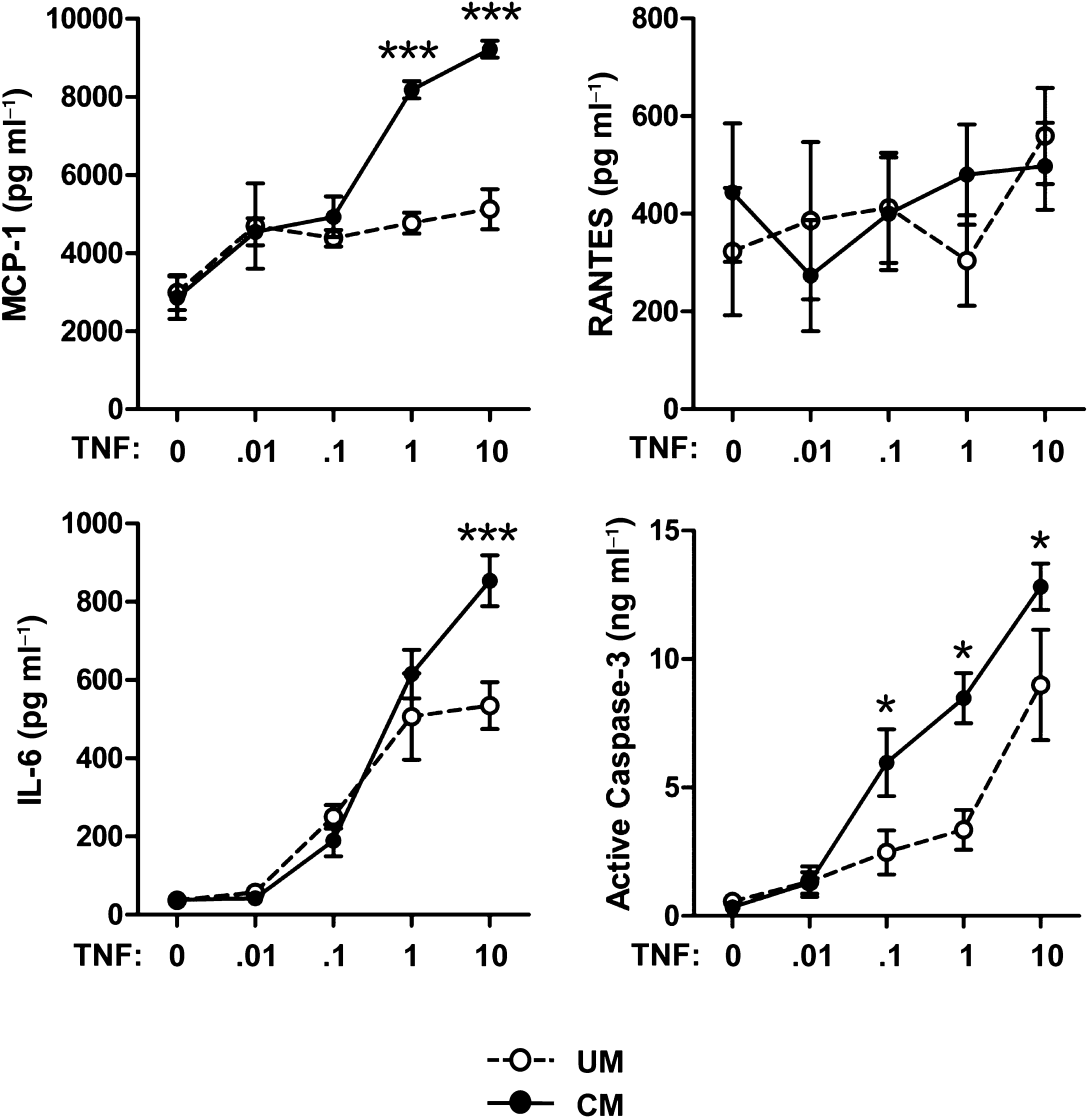
Comparison of TNF-induced production of MCP-1, RANTES, IL-6 and cleaved Caspase-3 between EC^UM^ and EC^CM^. Subcutaneous fat-derived EC from both patient categories were stimulated with different doses of TNF. In the case of MCP-1, RANTES and IL-6, supernatants were collected after 12 h of incubation and concentrations were measured by ELISA. For activated Caspase-3, supernatants and cell extracts were pooled before analysis by ELISA. Results are expressed as mean ± SD of three determinations in two experiments (Dunn's test, *P < 0.05 and ***P < 0.001).

To determine whether the release of MCP-1, RANTES and IL-6 by EC^UM^ and EC^CM^ varied upon inflammatory stimulus, we performed a series of ELISA in the presence of increasing concentrations of TNF, ranging from 0.01 to 10 ng ml^−1^. The release of both MCP-1 and IL-6 was induced by TNF in EC^UM^ and EC^CM^ and this effect was dose-dependent. Significantly higher levels of MCP-1 and IL-6 were produced by EC^UM^ than by EC^CM^ when TNF concentrations reached 1 and 10 ng ml^−1^ respectively (Fig. 4). Conversely, there was no significant effect of TNF on release of RANTES by either cell types, nor was there a clear tendency or statistically significant difference in its production between EC^CM^ and EC^UM^ (Fig. 4).

### Activation of caspase-3 in TNF-stimulated EC^CM^ and EC^UM^

The level of activated caspase-3 in EC^CM^ than in EC^UM^ was clearly modulated by the amount of cytokine added to the medium. Caspase-3 activation was significantly higher in EC^CM^ than in EC^UM^ at TNF concentrations of 0.1 ng ml^−1^ and above (*P* < 0.05).

## Discussion

A central component of CM pathophysiology appears to be the activation of microvascular EC, resulting both from the cytoadherence of infected erythrocytes to EC and from the pro-inflammatory effects of locally released cytokines (Wassmer *et al*., 2003). The consequences of this endothelial inflammation are numerous and include: (i) the upregulation of endothelial receptors, enhancing PRBC sequestration, (ii) an increased shedding of endothelial MP, (iii) further release of cytokines and chemokines, and (iv) the trigger of a TNF-dependent pro-apoptotic pathway (as reviewed in Combes *et al*., 2006). Since brain microvascular EC are profoundly affected in these ways by TNF, variation in the responsiveness of EC to TNF in different individuals could be a factor affecting the severity of disease in patients with malaria.

Several studies indicate that microvascular EC differ from large vascular EC in morphology or function (Grau and Lou, 1993), and EC derived from different organs also differ in response to some cytokines (Belloni *et al*., 1992). In addition, there are considerable variations in both adhesion molecule expression and the functional properties of EC depending on their position within the vascular bed of a particular tissue (Ley *et al*., 1992). To examine the possibility that our findings with cultured subcutaneous fat-derived EC might not reflect the properties of intracerebral vascular EC, we took the opportunity to examine EC from both brain and subcutaneous fat in patients dying of CM who came to autopsy. Both EC types exhibited similar endothelial features. Cells of both origins were shown to express major pro-inflammatory cytokine-induced endothelial adhesion molecules such as VCAM-1 and ICAM-1 (Springer, 1990), which were upregulated to a similar degree upon activation by TNF (Table 1). Two discrepancies confirming previously published data were observed between subcutaneous fat- and brain-derived EC^CM^: the former expressed CD36 and not ZO-1 whereas the latter expressed ZO-1 but not CD36. ZO-1 plays a crucial role in the permeability of the blood–brain barrier (Staddon *et al*., 1995), and its presence has been widely acknowledged as a hallmark of adjacent brain EC (Collins *et al*., 2006). In contrast, the expression of CD36, a non-inducible receptor for PRBC, has been reported to be low and irregular in cerebral microvasculature in several studies (Turner *et al*., 1994), as opposed to microvascular beds from subcutaneous and visceral adipose tissue (Bonen *et al*., 2006). However, these two differences were irrelevant to our study since variation in EC monolayer permeability was not one of our read-out parameters and CD36 expression is not regulated by TNF. Our results show that EC cultured from subcutaneous fat closely resemble cerebral vascular EC in several properties relevant to the pathogenesis of malaria, which suggests they may be useful in an *ex vivo* model.

Making use of this model, we have shown that subcutaneous fat-derived EC^CM^ and EC^UM^ display significantly different *ex vivo* responsiveness to TNF. We demonstrated that, when compared with EC^UM^ responses, EC^CM^ express significantly higher levels of ICAM-1, VCAM-1 and CD61, produce more endothelial MP, MCP-1 and IL-6 and are more prone to undergo apoptosis upon stimulation with TNF.

While sequestration of PRBC in the brain has been widely described in malaria, its association with disease severity and especially with CM is still debated. Specific binding of PRBC to ICAM-1 has been reported to be higher in CM patients compared with patients with uncomplicated disease (Newbold *et al*., 1997). Our results are in line with the former, and indicate that EC^CM^ upregulate much higher levels of ICAM-1 on their surface than EC^UM^, providing an increased number of PRBC binding sites. This inter-individual difference in ICAM-1 upregulation upon TNF stimulation has also been described in human umbilical vein EC (HUVEC). In a recently published study, Beck and colleagues isolated 30 lines of HUVEC and upon stimulation with TNF, some EC, i.e. type I responders, displayed a low ICAM-1 upregulation while type II responders exhibited a significantly higher level of the receptor (Beck *et al*., 2006). However, we cannot exclude the possibility of a long-lasting effect of malaria parasite-derived glycosylphosphatidylinositol, which has been reported to alter cell-surface expression of various adhesion molecules on HUVEC (Schofield *et al*., 1996). Such stimulation could well alter the progeny of cells derived from patients with severe malaria despite their time in culture.

In addition to ICAM-1, VCAM-1 and CD61 upregulation was also shown to be more pronounced in EC^CM^ compared with EC^UM^. Since VCAM-1 acts as a receptor for PRBC (Ockenhouse *et al*., 1992), this phenomenon might also contribute to the amplification of sequestration in CM patients. The upregulation of CD61, which is involved in the binding of activated platelets to EC, may account for the phenomenon of platelet accumulation in cerebral microvessels, a histological feature of fatal CM (for review see Moxon *et al*., 2009). However, no commensurate upregulation of CD51, its pairing beta integrin chain to form the α_v_β_3_ receptor, was measured and the significance of this finding will require further investigation.

Inter-individual differences in the endothelial inflammatory response to TNF in our study also included the production of membrane MP. A recent study in Malawian children demonstrated significantly higher levels of circulating endothelial MP during the acute phase of CM compared with levels observed in parasitaemic controls. Levels of endothelial MP correlated positively with plasma levels of TNF and returned to normal following recovery (Combes *et al*., 2004). In the present study, *in vitro* cultures of EC^CM^ released significantly higher numbers of MP when exposed to TNF than did cultures of EC^UM^. We do not know whether this increased release plays a role in the cascade of events leading to CM, but recent analyses in a mouse model provided evidence for both pro-inflammatory and pro-coagulant effects of MP (Combes *et al*., 2005), suggesting that MP could potentially enhance the severity of intracerebral pathology.

In addition to monocytes, the vascular endothelium is a major source from which circulating cytokines and chemokines can be derived, especially in inflammatory conditions. This is particularly true for IL-6, MCP-1 and RANTES (Krishnaswamy *et al*., 1999). Using the *ex vivo* model we showed that EC^UM^ release less MCP-1 and IL-6 under TNF stimulation than EC^CM^, while the release of RANTES is similar in both groups. MCP-1 is a CC chemokine produced by macrophages and EC in response to diverse stimuli, including TNF (Murao *et al*., 1999), attracting both monocytes and lymphocytes (Carr *et al*., 1994). Mononuclear leucocytes have been identified among the sequestered cells in brain venules in fatal paediatric CM (Grau *et al*., 2003), and monocytes expressing the appropriate ligand can bind to ICAM-1. Local MCP-1 release coupled to upregulation of ICAM-1 might together be responsible for both the recruitment and the binding of monocytes on cerebral microvasculature (Beekhuizen *et al*., 1990).

Like MCP-1, RANTES is a chemokine that can be released by EC under a pro-inflammatory stimulus, but there was no difference in RANTES production in cells from both patient categories. These results suggest a selective triggering by TNF of the expression and secretion of MCP-1 but not of RANTES, similar to the one reported in studies of human herpesvirus 8-infected HUVEC cultures (Caselli *et al*., 2007). Alternatively, there may be a post-transcriptional inhibition of RANTES release. Our findings are in keeping with the reported observation that low plasma levels of RANTES are associated with mortality in children with CM (John *et al*., 2006).

The higher level of IL-6 by EC^CM^ than EC^UM^ observed in this study is in line with data showing that elevated plasma levels of IL-6 are associated with severe malaria (Larkin *et al*., 2009). IL-6 together with TNF can increase cerebral EC permeability (Duchini *et al*., 1996), which could contribute to the cerebral oedema observed in CM (Patnaik *et al*., 1994). IL-6 might also aggravate PRBC and leucocyte cytoadherence by upregulating ICAM-1 and VCAM-1 on the EC surface (Watson *et al*., 1996). IL-6 has been reported to inhibit the ability of plasma enzyme ADAMTS13 to cleave full-length VWF (Bernardo *et al*., 2004), possibly leading to procoagulant events characteristically seen in severe malaria, including the adhesion and clumping of platelets to circulating or endothelial surface-anchored ultra large VWF multimers (Bridges *et al*., 2010).

The induction of endothelial apoptosis during CM has been widely described in both *in vitro* (Wassmer *et al*., 2006) and murine models of the pathology (Wiese *et al*., 2006). Its occurrence *in vivo* during human CM, however, remains debated. In the present study we observed a significantly higher level of caspase-3 cleavage in EC^CM^ compared with EC^UM^, which is consistent with previous results. However, since circulating levels of the cytokine rarely exceed 1 pg ml^−1^ during human malaria infection, the induction of apoptosis is likely to involve a massive local release of TNF in areas of intense sequestration. This is in line with previous reports of focal accumulation of TNF in the brain of patients who died of CM (Udomsangpetch *et al*., 1997). Whether these findings accurately reflect the completion of the whole pro-apoptotic cascade *in vivo* is not known.

Taken together, our findings demonstrate a difference in responsiveness to TNF between EC derived from CM patients and those derived from UM patients. One explanation for this difference might be that EC responsiveness *in vitro* is affected by the severity of disease in the patient from whom the EC were collected. This explanation is unconvincing, because the EC with which we worked had been cultured through several cell cycles during a period of up to 3 weeks. Any influence of conditions prevailing *in vivo* is unlikely to have persisted throughout this period. An alternative explanation is that there is an inherent diversity among individuals in the responsiveness of their vascular EC to pro-inflammatory stimuli. This might result from (i) a variation in TNF ‘intake’ by the cells, (ii) a long-term effect due to specific *P. falciparum* exposure, (iii) a genetic inter-individual variation in reactivity to inflammation or (iv) a combination of all the factors. The differences we observed between EC^CM^ and EC^UM^ would then suggest that greater antecedent EC responsiveness predisposed some individuals to the development of more severe disease. Inherent diversity of individuals in the responsiveness of their EC to TNF stimulation has recently been reported by Beck and colleagues in a study of cultured lines of HUVEC (Beck *et al*., 2006) and in the fever effect on PRBC cytoadherence in individual HUVEC isolated from Kenyan mothers (A.G. Craig, unpubl. data). In addition, a recent study in Ugandan children and Thai adults revealed that TNF levels do not clearly discriminate between UM and CM patients (Lovegrove *et al*., 2009), suggesting that a differential responsiveness to this cytokine rather than a differential production might account for the clinical outcome of malaria infection.

## Experimental procedures

### Patients

We studied cases of UM and CM admitted to the paediatric research ward of the Queen Elizabeth Central Hospital, Blantyre, Malawi between January 2007 and August 2009. All patients with UM (*n* = 6; four males and two females; mean age 4.7 years, mean parasitaemia 53 349 parasites µl^−1^, parasitaemia range 151 500) had *P. falciparum* parasitaemia and a packed cell volume above 25% and were fully conscious (Blantyre coma score 5/5). UM patients' relatives gave fully informed consent for a subcutaneous fat tissue sample to be taken. This was performed by needle aspiration using a hollow bore ‘menghini’ needle on the upper external thigh after application of local anaesthetic cream for 2 h to temporarily numb the surface of the skin (EMLA Cream 5%, AstraZeneca). Patients with CM (*n* = 7; five males and two females; mean age 3.3 years, mean parasitaemia 43 141 parasites µl^−1^, parasitaemia range 90 724) were admitted to the hospital in coma (Blantyre coma score 2/5 or less), had *P. falciparum* parasitaemia with no other clinically evident cause of unconsciousness and were found to have malarial retinopathy (for review, see Beare *et al*., 2006). They were treated with quinine, as described elsewhere (Taylor *et al*., 2004). Two patients who recovered had subcutaneous fat tissue sample collected in the ward as described above. Five patients died of CM and once consent was granted from parents or guardians (Taylor *et al*., 2004), a full autopsy was conducted at the mortuary of the Queen Elizabeth Central Hospital, during which brain and subcutaneous fat tissue samples were collected (approximately 5 mm^3^). The autopsy or the subsequent histological studies confirmed the diagnosis for these five CM patients. This study was conducted according to the principles expressed in the Declaration of Helsinki and was approved by the ethical review committees of the College of Medicine, University of Malawi, the Liverpool School of Tropical Medicine and Michigan State University. The parents, relatives or guardians of all patients provided written informed consent for the collection of samples and subsequent analysis.

### Isolation, purification and culture of EC derived from patients

**Fig. 5:**
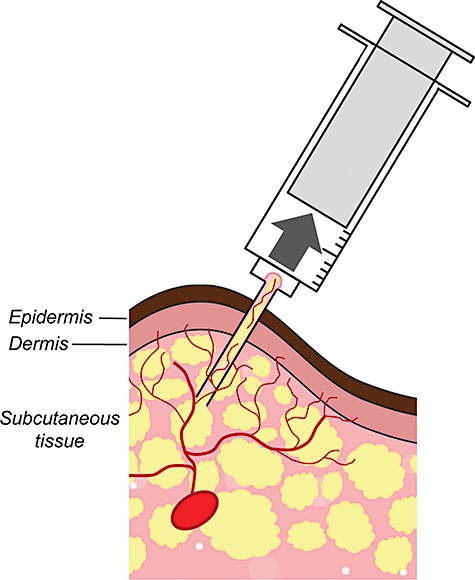
Schematic representation of needle aspiration biopsy. The diagram shows the technique used to isolate subcutaneous fat-derived fat EC by inserting a hollow bore ‘menghini’ needle in the upper external thigh of patients with malaria (UM and CM) after application of a local anaesthetic for 2 h. A strong manual aspiration force represented by the arrow allowed the sampling of approximately 5 mm^3^ of adipose tissue, from which EC were isolated, selected and cultured before analysis.

**Fig. 6:**
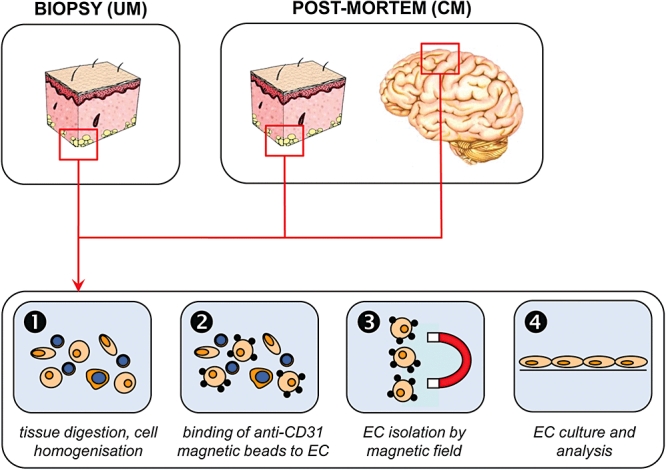
Method for sampling and isolating EC^CM^ from brain and subcutaneous fat tissue. EC^CM^ were collected at autopsy and isolated from both subcutaneous fat and brain tissue from patients who died of CM (*n* = 7). Brain EC^CM^ were only used for a phenotypical comparison with subcutaneous fat-derived EC^CM^ in the first part of our study to assess the relevance of the latter as an accurate model of cerebral endothelium.

Subcutaneous fat tissue-derived EC were obtained from all patients (fatal CM at autopsy and UM in the ward, Fig. 5) while brain EC were only obtained in fatal CM cases (Fig. 6). For clarity, EC derived from CM patients will be referred to as EC^CM^ and EC derived from UM patients as EC^UM^. Cells were isolated following a protocol adapted from Hutley *et al*. (2001) as described below.

#### Isolation

Briefly, biopsies were transported to the laboratory in PBS and transferred to gentleMACS™ Dissociator C-tubes (Miltenyi Biotec). Both brain and subcutaneous tissues were treated as follows: (i) samples were finely minced and incubated for 15 min at 37°C in digest solution (in mM: 25 Hepes, 5 glucose, 120 sodium chloride, 50 potassium chloride and 1 calcium chloride) containing 3 mg ml^−1^ type II collagenase and 1.5% bovine serum albumin (BSA). The ratio of digest solution to tissue was 4:1. C-tubes were then connected to the gentleMACS™ Dissociator and spun for 60 s before being incubated for 15 additional minutes at 37°C. The operation was repeated three times. The resultant digest material was filtered through a 250 µm mesh (Sefar), and adipocytes and free oil were separated from the stromovascular components by centrifugation at 250 *g* for 5 min at 4°C. (ii) The stromovascular pellet was resuspended, washed and centrifuged in DPBS containing 10% BSA (500 *g*, 5 min, 4°C). This was repeated and followed by a final wash in DPBS alone. (iii) The resulting pellet was incubated in 0.25% trypsin containing 1 mM EDTA (Invitrogen) for 15 min at room temperature with gentle automated agitation. Trypsin was neutralized by addition of Hanks' balanced salt solution (HBSS) containing 5% fetal bovine serum (FBS; Sigma). (iv) Large fragments of connective tissue were then removed by filtration through 100 µm mesh (Sefar). (v) The filtrate was centrifuged (500 *g*, 5 min, 4°C), and the pellet was resuspended and plated into 1% gelatine-coated 25 cm^2^ culture flasks (Corning) in DMEM-F12 medium (Invitrogen) supplemented with EGM-2 MV SingleQuots (Lonza), 150 µg ml^−1^ EC growth supplement (ECGS, Sigma) and 30 ng ml^−1^β-EC growth factor (β-ECGF). This mixed cell population was cultured for 3–5 days at 37°C in 5% CO_2_.

#### Selection of microvascular endothelial cells (MVEC) with anti-CD31 Dynabeads

After a culture period of 7–10 days, the cells were incubated with Trypsin-EDTA for 4–5 min at 37°C, followed by neutralization of trypsin with HBSS containing 5% FBS and centrifugation. The pelleted cells were resuspended in 1 ml of PBS containing 0.1% FBS and incubated with 50 µl of anti-CD31-coated Dynabeads under gentle agitation (20 min, 4°C). The cell/bead suspension was brought to a total volume of 10 ml with 0.1% FBS in PBS, and EC were selected using a magnetic particle concentrator for 3 min at room temperature. With the tube still in the magnet, non-selected cells were discarded and EC were then washed with a further 10 ml of PBS containing 0.1% FBS and reselected using the magnetic particle concentrator (3 min). This wash/selection procedure was repeated five times. Selected cells (EC) were plated on to 1% gelatin-coated culture flask in EC growth medium as described above.

#### Cell culture

Cells were maintained at 37°C in an atmosphere of 5% CO2. The medium was changed every 2–3 days. As EC numbers increased, the concentration of β-ECGF in the growth medium was decreased from 30 to 10 ng ml^−1^. Brain and subcutaneous fat tissue-derived EC were used in experimental work between *passages 2* and *4*.

### Characterization and phenotypic comparison of subcutaneous fat and brain-derived EC^CM^

In order to assess the relevance of subcutaneous fat-derived EC as a representative model for cerebral endothelium, cells obtained from brain and adipose tissue at autopsy were characterized and compared as described below.

#### Morphology

Cultures were examined by inverted phase-contrast microscopy for the characteristic cobblestone-like morphology of EC.

#### Immunofluorescence

Confluent EC were rinsed with PBS, fixed with 1% w/v of paraformaldehyde (PFA) for 15 min on ice, washed with PBS and incubated with FITC-coupled mouse anti-human CD31, CD54, CD36 (PECAM-1, clone 5.6E; ICAM-1, clone 84H10 and clone FA6.152, Beckman Coulter Immunotech), mouse anti-human VWF (clone F6/86, DAKO Cytomation) revealed by a secondary goat anti-mouse Alexa488® -coupled mAb (Molecular Probes) or FITC-coupled anti-ZO-1 (clone ZO-1-1A12, Zymed Laboratory). A non-specific isotype-matched mouse IgG1 (Beckman-Coulter Immunotech) was used for all controls. The expression of E-selectin (CD62E) was also investigated by use of a monoclonal antibody (clone CL2/6, Serotec) revealed by a secondary goat anti-mouse Alexa594®-coupled mAb (Molecular Probes). In this case, the immunofluorescence analysis was performed with cells pre-treated for 6 h with TNF (10 ng ml^−1^, Sigma).

#### Flow cytometry

Confluent monolayers of EC were stimulated, or not, with TNF (overnight or 6 h, 10 ng ml^−1^) before analysis. EC were then harvested by a short trypsin-EDTA treatment, washed, and labelled by indirect labelling using mouse anti-human CD106, CD54, CD51, CD61, CD31, CD36 (Beckman-Coulter Immunotech), CD40 (Diaclone), CD62E and CD146 (Serotec) mAbs as first step (as detailed in Table 1) and then with secondary goat anti-mouse Alexa488® -coupled mAb (Molecular Probes, Eugene, USA). A non-specific isotype-matched mouse IgG1 (Beckman-Coulter Immunotech) was used for all controls. Cells were then resuspended in PBS before flow cytometry analysis on a FACScalibur (Becton Dickinson). *Alexa488*® signal was detected in the FL1 channel (488 nm laser and 530/30 band-pass filter). The area corresponding to EC was defined, and mean fluorescence intensities (MFI) of the positive cell populations were measured for each antigen.

### Determination of adhesion molecules expression by TNF-activated EC^UM^ and EC^CM^ using cell-based ELISA and flow cytometry

Endothelial cells were seeded on 1% gelatin pre-coated flat-bottom 96-well plates (10^4^ cells well^−1^) and grown to confluence. Cells were then washed with DMEM-F12 medium and stimulated overnight separately with increasing concentrations of TNF (0, 0.01, 0.1, 1 and 10 ng ml^−1^). After stimulation, cells were washed a second time with DMEM-F12, fixed with −20°C methanol for 15 min at room temperature and incubated with PBS containing 5% FCS and 0.05% Tween (Merck) to block non-specific binding. Cells were then incubated, respectively, with monoclonal antibodies to human ICAM-1, VCAM-1, CD61 (Beckman-Coulter Immunotech) and CD62-E (Serotec), all at 10 µg ml^−1^ for 40 min at room temperature. For ELISA, the plate was then washed three times in PBS/Tween 20 and incubated for 45 min under mild shaking with 1 mg ml^−1^ goat anti-mouse IgG alkaline phosphatase conjugate (Promega). Cells were then washed twice with HBSS containing 5% BSA and once with 2.5 M diethanolamine (pH 9.5). Finally, the substrate solution was added, consisting of 0.58 mg ml^−1^ Attophos fluorescent substrate (Promega) and 2.4 mg ml^−1^ endogenous phosphatase activity blocking agent levamisole hydrochloride (Sigma), diluted in diethanolamine buffer. After 5 min, fluorescence was measured at an excitation wavelength 450 nm and an emission wavelength 580 nm in an automated ELISA reader. For flow cytometry analysis, cells were grown to confluence in 12-well plates and stimulated overnight with 10 ng ml^−1^ TNF. EC were then harvested by a short trypsin-EDTA treatment, washed, and labelled with monoclonal antibodies to human anti-ICAM-1, VCAM-1, CD61 (Beckman-Coulter Immunotech) and CD62-E (Serotec) as described above.

### Analysis of MP production by EC^UM^ and EC^CM^ by flow cytometry

Confluent EC^UM^ and EC^CM^ were left unstimulated or activated by TNF (1 and 10 ng ml^−1^) for 6 h before analysis. Culture supernatants were then collected and centrifuged at 1500 *g* for 15 min to discard EC and debris. Endothelial MP were labelled using annexin V-FITC and resuspended in binding buffer (Beckman-Coulter Immunotech) as previously described (Combes *et al*., 2004). MP present in supernatants were then quantified by flow cytometry.

### Quantification of MCP-1, RANTES and IL-6 release and caspase-3 activation levels by ELISA

Confluent cells were washed with DMEM-F12 medium and stimulated separately overnight with fresh culture medium containing increasing concentrations of TNF (0, 0.01, 0.1, 1 and 10 ng ml^−1^). For MCP-1, RANTES and IL-6, cell supernatants were harvested, centrifuged to remove cell debris and stored at −80°C. After thawing, samples were analysed for the presence of MCP-1, RANTES and IL-6 using specific quantitative enzyme-linked immunosorbent assays (ELISA) kits (R&D System Quantikine for MCP-1 and RANTES; Becton Dickinson OptEIA for IL-6). For active Caspase-3, cells were washed in PBS and cell extracts were prepared according to the manufacturer's instruction before being analysed immediately by ELISA (R&D System Quantikine). Each sample was tested in triplicate.

## Statistics

Statistical analyses were performed with STATA 8 ©. Data were analysed by the Kruskall–Wallis and Dunn's pairwise tests. Results are expressed as means ± standard deviations. A value of *P* < 0.05 was considered significant.
